# Management of disease-modifying therapies in multiple sclerosis and comorbid rheumatoid arthritis

**DOI:** 10.1186/s42466-025-00414-y

**Published:** 2025-07-17

**Authors:** Franz Felix Konen, Torsten Witte, Diana Ernst, David Hagin, Konstantin Fritz Jendretzky, Nora Möhn, Sandra Nay, Lea Grote-Levi, Kurt-Wolfram Sühs, Luisa Klotz, Steffen Pfeuffer, Refik Pul, Christoph Kleinschnitz, Marc Pawlitzki, Sven G. Meuth, Thomas Skripuletz

**Affiliations:** 1 Department of Neurology, Hannover Medical School, Carl-Neuberg-Straße 1, 30625 Hannover, Germany; 2 Department of Rheumatology and Clinical Immunology, Hannover Medical School, 30625 Hannover, Germany; 3 Allergy and Clinical Immunology Unit, Department of Medicine, Tel-Aviv Sourasky Medical Center and Sackler Faculty of Medicine, University of Tel Aviv, 6 Weizmann St, Tel-Aviv, 6423906 Israel; 4 Department of Neurology with Institute of Translational Neurology, University Hospital Muenster, 48149 Muenster, Germany; 5 Department of Neurology, University Hospital Giessen and Marburg, Justus-Liebig-University Giessen, 35392 Giessen, Germany; 6 Department of Neurology, Center for Translational Neuro- and Behavioral Sciences, University Medicine Essen, University Hospital Essen, Essen, Germany; 7 Department of Neurology, Medical Faculty, Heinrich Heine University Dusseldorf, 40225 Dusseldorf, Germany

**Keywords:** Multiple sclerosis, Rheumatoid arthritis, Disease-modifying therapy, Autoimmune disorders, Treatment, Beneficial and adverse effects, BTK inhibitor

## Abstract

**Background:**

Comorbid autoimmune disorders, including rheumatoid arthritis (RA), are common in people with multiple sclerosis (MS). Both conditions share pathogenic similarities, enabling potential overlap in treatments. While numerous disease-modifying therapies (DMT) are approved for MS and new options are under clinical trial, their effectiveness in RA varies.

**Main body:**

A PubMed literature review was conducted to evaluate the effects of approved and currently investigated MS-DMT on MS and RA and vice versa. Certain MS-DMT showed beneficial effects for RA, such as teriflunomide, anti-CD20 therapies, and cladribine, while others demonstrated no significant impact (type-I interferons, Bruton´s tyrosine kinase (BTK) inhibitors) or lacked trials (sphingosine-1-phosphate receptor modulators, glatiramer acetate). In contrast, BTK inhibitors were shown to be effective for inactive secondary progressive forms of MS, whereas secukinumab showed limited effects in relapsing MS. Concerning DMT for RA in MS, no significant benefit was observed for abatacept, and there are no trials for Janus kinase inhibitors, or interleukin-(IL)-6 receptor inhibitors (tocilizumab, sarilumab). Adverse events, including RA exacerbation, were reported for some MS-DMT like dimethyl fumarate, alemtuzumab, and natalizumab. Tumor necrosis factor alpha (TNFα) inhibitors increased disease activity in MS patients.

**Conclusion:**

Among approved DMT for MS and RA, teriflunomide and anti-CD20 therapies are the most suitable options for moderately or highly active MS with comorbid RA. Cladribine may also be considered, while TNFα inhibitors are contraindicated.

## Background

Multiple sclerosis (MS) is a chronic autoimmune mediated disease, which is characterized by demyelination of axons of the central nervous system (CNS) [1]. The incidence of comorbid autoimmune diseases is higher in people with MS than in healthy individuals, and up to 18% of all suffer from additional autoimmune comorbidities during their life [2, 3]. The most common of these are rheumatoid arthritis (RA), psoriasis, and inflammatory bowel diseases [2, 3]. MS and RA share several similarities in their pathogenesis such, as the important role of T lymphocytes and involvement of B lymphocytes as well as a genetic predisposition and familial clustering [1, 4, 5]. These similarities result in partly overlapping therapeutic options, allowing to address a comorbid RA in people with MS with only one treatment that beneficially affects both disease entities [6]. However, both disease entities also show differences in their pathogenesis and the role of different cytokines and chemokines [1, 4, 5]. These differences lead to potentially harmful treatment options in people who suffer from both RA and MS [6]. Exemplary, tumor necrosis alpha (TNFα) inhibitors serve as a useful treatment option for people with RA but potentially worsen the disease course in people with MS [7]. In the present review, we aim to provide an overview of the therapeutic effects of various approved and currently investigated MS-DMT which might also have beneficial effcts in RA and vice versa.

## Methods

**Fig. 1:**
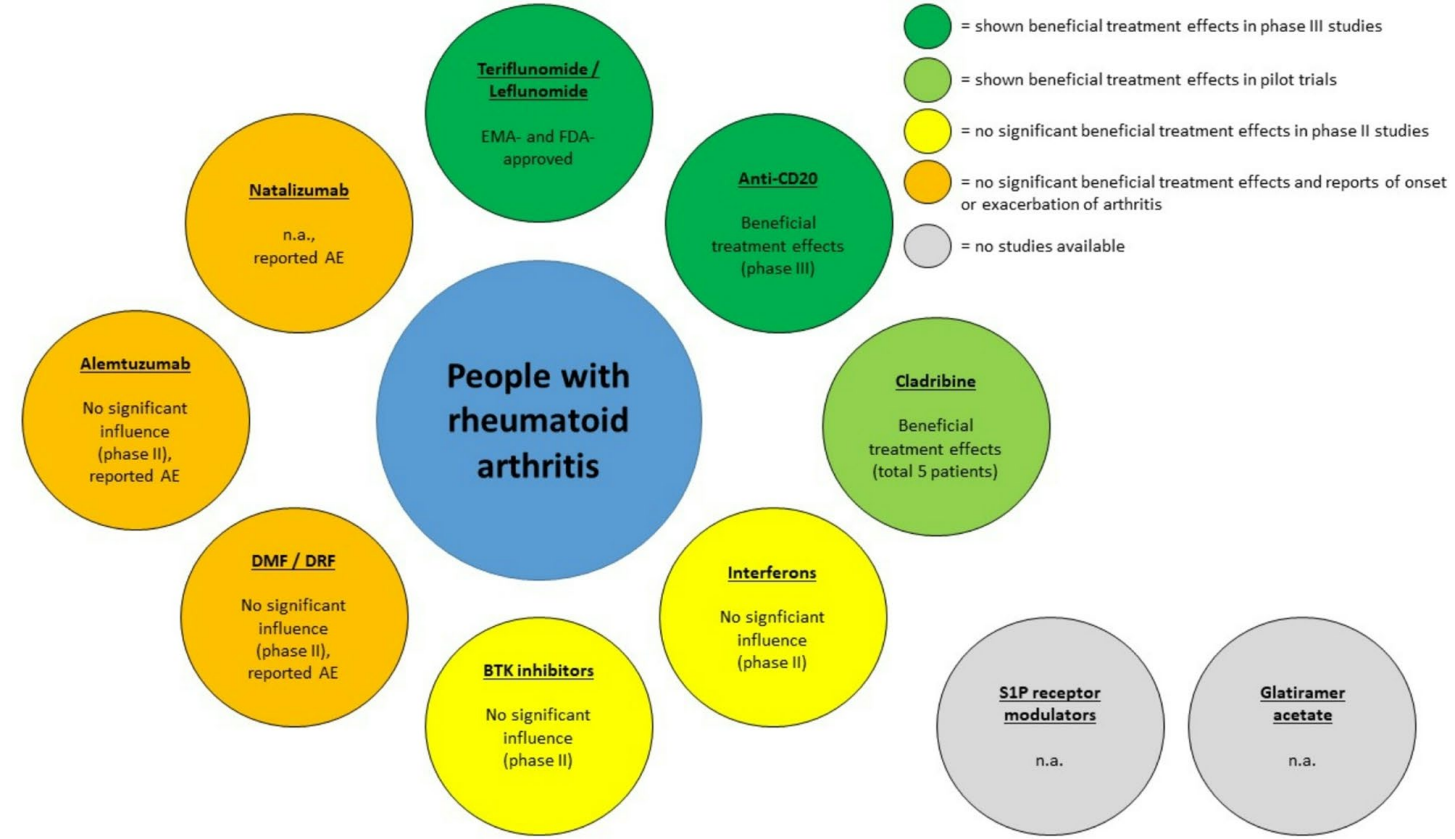
Disease-modifying therapies and those currently under investigation for MS, along with available data on treatment efficacy in rheumatoid arthritis. EMA = European medicin agency, FDA = food and drug administration, Anti-CD20 = anti-CD20-therapies (ocrelizumab, ofatumumab, ublituximab), BTK = Bruton´s tyrosine kinase, DMF = dimethylfumarate, DRF = diroximelfumarate, AE = adverse events (onset or exacerbation of arthritis), S1P = sphingosine-1-phosphate, n.a. = no reports in humans available

**Fig. 2:**
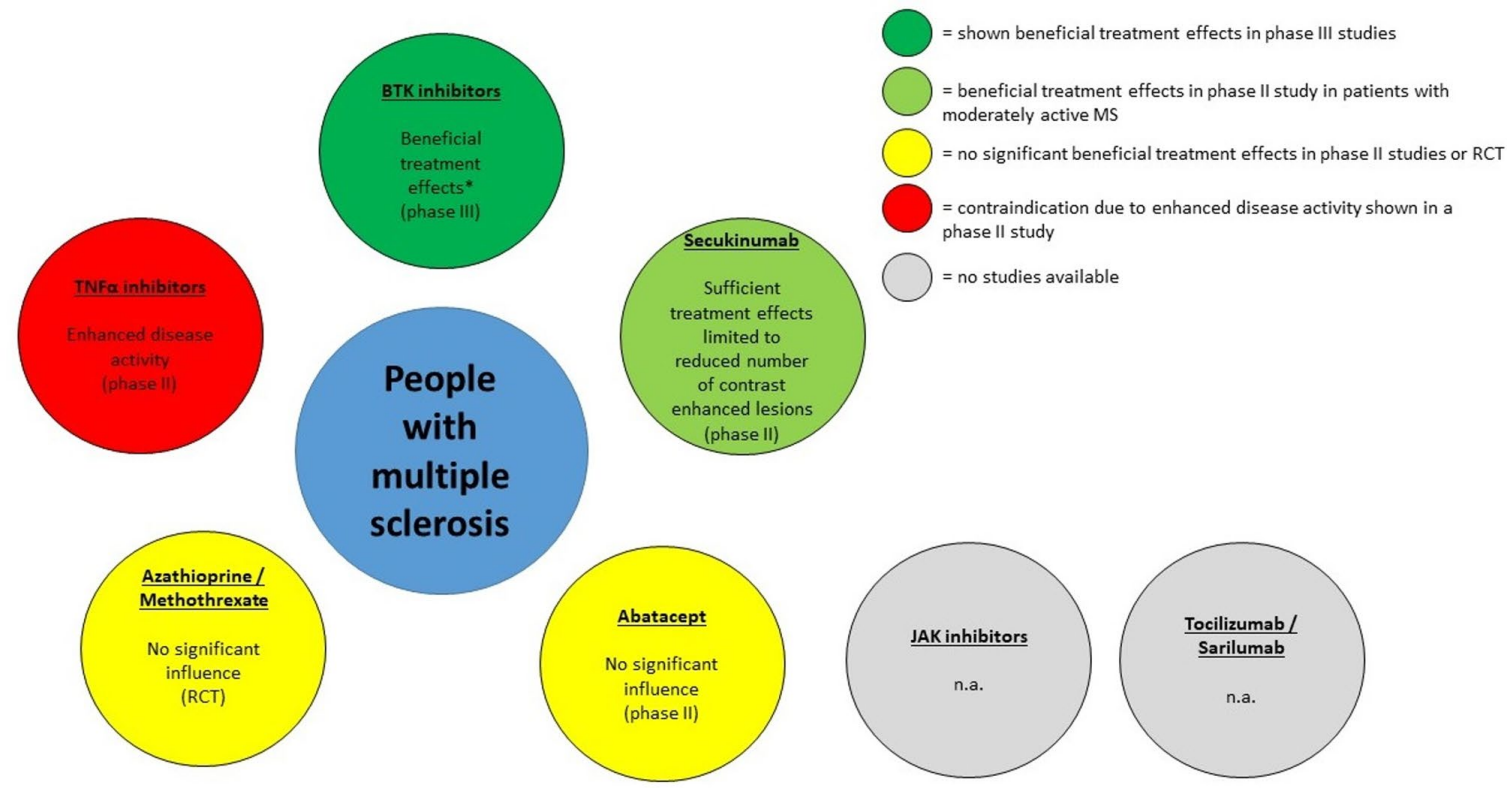
Disease-modifying therapies and those currently under investigation for rheumatoid arthritis, along with available data on treatment efficacy in MS. BTK = Bruton´s tyrosine kinase, * = progressive forms of MS, RCT = randomized controlled trial, JAK = Janus kinase, TNFα = tumor necrosis alpha, n.a. = no reports in humans available

**Fig. 3:**
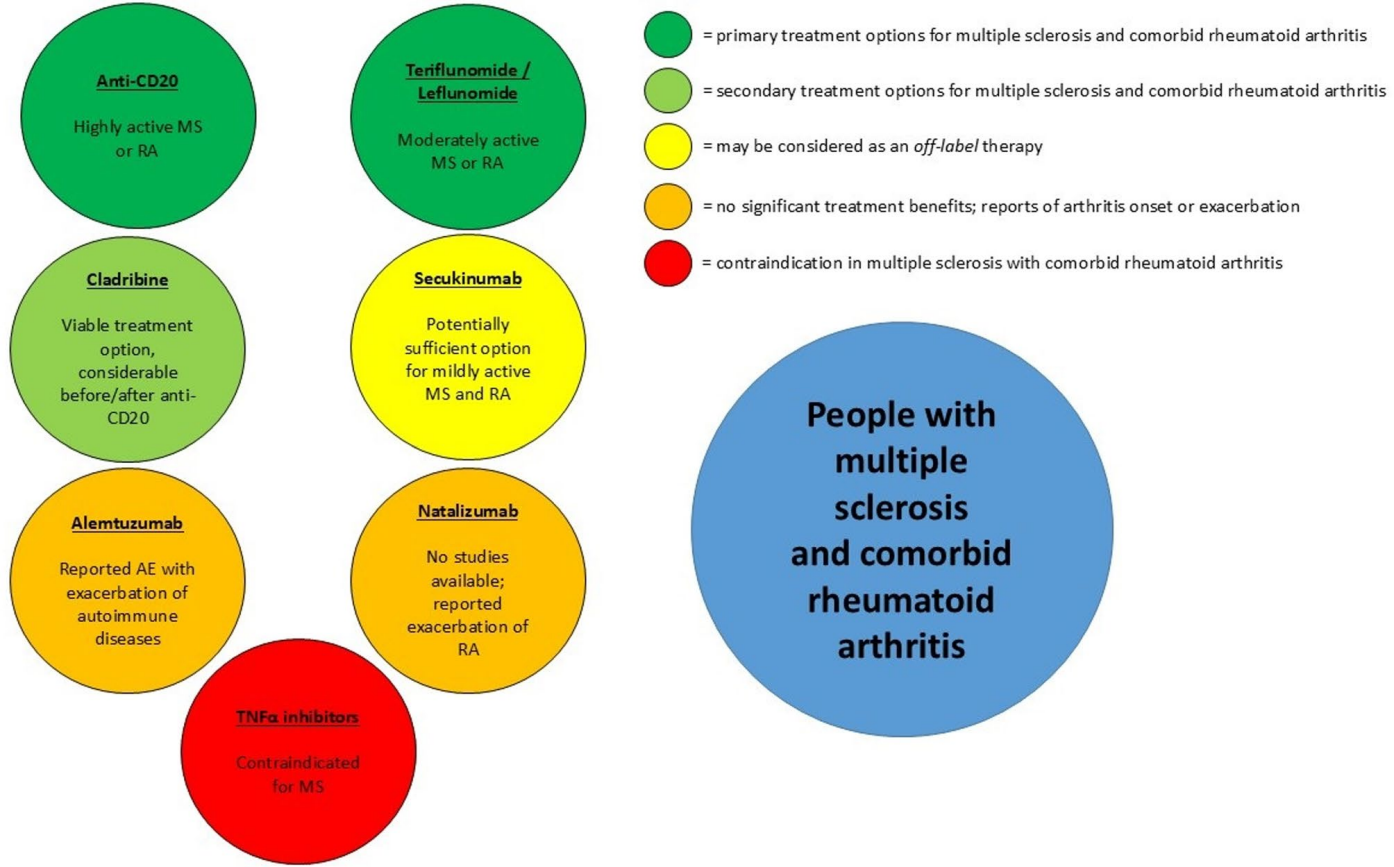
Considereable disease-modifying therapies for people with multiple sclerosis and comorbid rheumatoid arthritis. MS = multiple sclerosis, RA = rheumatoid arthritis, anti-CD20 = anti-CD20-therapies (ocrelizumab, ofatumumab, ublituximab), AE = adverse events (onset or exacerbation of arthritis)

Literature research (NIH National Library of Medline PubMed.gov database, https://pubmed.ncbi.nlm.nih.gov/) was conducted until December 2024 to describe the effects of different approved or investigated MS-DMT on MS and RA and vice versa. Search terms were the respective DMT for MS or RA and “multiple sclerosis” or “rheumatoid arthritis” (for example “ocrelizumab AND rheumatoid arthritis”, “tocilizumab AND multiple sclerosis”). More than 500 publications were screened for eligibility. Those articles describing treatment effects of the respective DMT in case reports, case series, prospective and retrospective observational studies, uncontrolled clinical trials, randomized controlled trials (RCT), as well as phase I– III studies were considered suitable for this review article. Excluded publications were duplicates, those without description of treatment effects (for example only adverse events) and unclear description of included patients. The results of this comprehensive literature search on approved or currently investigated DMT for MS and RA in the respective other indication are summarized in Figs. 1, 2 and 3.

Our review refers to the current status of approval by European Medicine Agency (EMA) and Food and Drug Administration (FDA). All MS-DMT, including their dosages, therapeutic regimens, or formulations used in MS, are not approved for the treatment of RA. Therefore, this review cannot recommend specific in-label treatment for comorbid RA in people with MS, but rather provides information on beneficial or adverse effects of a particular drug based on reports from the literature.

In the following, results were categorised according to the expected treatment effects for MS and comorbid RA also considering emerging therapies into beneficial treatment options, treatment options with insufficient beneficial effects on MS or RA, treatment options with lack of evidence in MS or RA, treatment options without beneficial effects and reported adverse events in MS or RA, and finally, treatment options that should be avoided due to known potentially harmful effects.

## Results

### Pathogenesis of MS and RA

The pathogenesis of RA involves a complex interplay of genetic and environmental factors that result in dysregulated immune responses [4]. Key genetic contributors include specific HLA-DR alleles, particularly HLA-DRB1 with a shared epitope, and other immune regulation risk alleles, which are associated with T cell activation [4]. CD4^+^ T cells, particularly the T helper (Th) 1 and Th17 subsets, are critical mediators of inflammation in RA and activate macrophages and synovial fibroblasts through release of inflammatory cytokines such as TNF-α, interleukin- (IL)-6, and IL-17 [4]. These cytokines are crucial in driving the inflammatory cascade, joint destruction, and the recruitment and activation of other inflammatory cells, thereby sustaining synovitis [4]. Activated macrophages are abundant in the synovial lining and secrete pro-inflammatory cytokines, fibroblast-like synoviocytes which act hyperproliferative, and produce matrix-degrading enzymes that contribute to cartilage destruction, and antibody-producing B cells [4]. Antibodies such as rheumatoid factor (RF) and anti-citrullinated protein antibodies (ACPAs) may even precede clinical disease and contribute to joint inflammation and damage [4]. Beyond antibody production, B cells contribute to immune complex formation and cytokine secretion, further amplifying the inflammatory response [4].

Since RA and MS are both autoimmune mediated diseases, they share several similarities in their pathogenesis. In terms of genetics, both conditions are associated with specific HLA-DR alleles [1, 4]. In MS, HLA-DRB1*15:01 is strongly linked to disease susceptibility, similar to the shared epitope of HLA-DR alleles in RA [1, 4]. CD4^+^ T cells play a central role in both diseases, with Th1 and Th17 subsets being critical mediators of inflammation [1, 4, 5]. In addition, B cells and antibody production are involved in the pathogenesis of both MS and RA [1, 4, 5]. Despite these similarities, there are significant differences between the two diseases. The primary distinction lies in the affected organs and effector cells, which are located in different compartments. Another major difference, though not fully understood, concerns the role of inflammatory cytokines such as TNF-α and IL-6 [1, 4, 5, 7]. In both MS and RA, TNF-α and IL-6 were found to be elevated and associated with pathogenesis, leading to the approval of anti-TNF-α and IL-6 receptor antagonists for the treatment of RA [1, 4–7]. However, in MS, these cytokines also appear to be involved in neuroprotection and tissue repair, making them challenging therapeutic targets for MS treatment, as further described in the sections below [7].

### Beneficial treatment options for MS and comorbid RA

#### Teriflunomide/leflunomide

Teriflunomide has gained regulatory approval for the management of relapsing MS at a prescribed dosage of 14 mg/day [8]. Leflunomide, the precursor of teriflunomide, which undergoes activation in plasma and intestinal mucosa following oral administration, has been granted approval by both the EMA and the FDA for the treatment of RA [8, 9]. Leflunomide dosing regimen for RA entails an initial loading dose of 100 mg/day for three days, followed by a daily dose ranging from 10 to 20 mg [8, 9]. The therapeutic mechanisms of teriflunomide involve reversible inhibition of dihydroorotate dehydrogenase (DHODH), an enzyme located in the mitochondria [10]. This inhibition leads to a reduction in pyrimidine de novo synthesis, subsequently resulting in diminished activation and proliferation of both B and T lymphocytes [10]. Furthermore, it results in an inhibition of mitochondrial respiratory activity with a preferential inhibition of this pathway in metabolically highly active T cells after high-affinity versus low-affinity antigenic stimulation [10].

Lastly, a recent phase II trial examined a second-generation DHODH inhibitor, vidofludimus, in comparison with placebo for the treatment of RA alongside methotrexate therapy [11]. The safety profile of vidofludimus resembled that of placebo and the primary efficacy endpoints were not met [11]. For the treatment of MS, a recent phase II study showed that compared with placebo, vidofludimus suppressed the development of new brain lesions with daily doses of 30 mg and 45 mg among adults with active relapsing-remitting MS (RRMS) and at least one gadolinium-enhancing cerebral MRI-lesion in the past 6 months [12].

Since leflunomide, the precursor of teriflunomide, is already approved for the treatment of RA, either drugs seem to be a valid therapeutic option for people with MS and comorbid RA.

#### Anti-CD20-antibodies (ocrelizumab/ofatumumab/ublituximab/rituximab)

Rituximab, ocrelizumab, ofatumumab and ublituximab are monoclonal antibodies targeting distinct epitopes on the CD20 receptor, resulting in the depletion of B cells and potentially a specific subset of CD20^+^ T lymphocytes [13]. Ocrelizumab has exhibited efficacy in treating relapsing MS and notably gained approval not only for relapsing MS but also as the first treatment for primary progressive multiple sclerosis [14]. Its administration involves intravenous infusion (600 mg every 6 months following two 300 mg induction doses within two weeks, or as recently EMA- and FDA-approved 920 mg subcutaneous every 6 months). Ofatumumab, on the other hand, is administered subcutaneously and secured approval for the management of relapsing MS (RMS) after ocrelizumab. The induction phase includes three weekly administrations of 20 mg, followed by a monthly dose of 20 mg [15]. Lastly, ublituximab was approved by the FDA and EMA for the treatment of RMS and is applied intravenously with an infusion regimen similar to that of ocrelizumab (450 mg every 24 weeks following an induction with 150 mg initially and 450 mg after two weeks) [16]. In scandinavian countries, rituximab is frequently used in MS patients and has shown beneficial treatment results in different large studies [17].

Currently, ocrelizumab, ofatumumab, and ublituximab are not approved for the treatment of RA.

Numerous preceding clinical studies and different phase III trials have demonstrated both clinical and serological improvements in RA with ocrelizumab treatment [18]. Differing from the MS treatment regimen, ocrelizumab dosages of 200 mg and 500 mg were administered at specific intervals [18]. Both dosages met primary endpoints, yet the 500 mg dose led to a higher incidence of severe adverse events [18]. Clinical amelioration of arthritic symptoms in people with RA was also evident in a phase II extension trial and two phase III trials for both subcutaneous and intravenous ofatumumab treatment [19]. The subcutaneous administration of 30 mg led to prolonged CD20 depletion following a single dose, with partial repletion observed in some patients, suggesting a treatment approach similar to that applied in MS cases [19]. In contrast, no clinical trials involving people with RA have been enrolled for ublituximab.

Since another anti-CD20-antibody (rituximab) is EMA- and FDA-approved for the treatment of RA and has shown beneficial treatment results in patients with MS, and ocrelizumab as well as ofatumumab showed beneficial treatment effects in phase III studies, anti-CD20-therapies seem to be a valuable treatment option on people with MS and comorbid RA.

#### Cladribine

Cladribine functions as a pro-drug, which upon phosphorylation becomes activated into the active metabolite (2-chlorodeoxyadenosine triphosphate, 2-CdATP) [20]. Operating as a purine analogue, it triggers apoptosis in both B and T lymphocytes. Its regulatory approval as a highly efficacious treatment for RMS is based on its ability to induce immune modulation. Cladribine is administered orally, following a specific treatment protocol that limits its use to a maximum of 20 days of tablets in the first two years of the total four year treatment regimen [21]. A non-controlled clinical trial involving five patients documented the use of subcutaneous cladribine for refractory RA [22]. This case series showed regression of clinical symptoms without changes in serologic parameters [22].

Although data on the use of oral cladribine in patients with RA is scarce, due to the relatively broad mode of action affecting B and T lymphocytes, usage of cladribine in people with MS and comorbid RA seems to be a reasonable therapeutic option.

### Treatment options with insufficient beneficial effects on MS or RA

#### Type I interferons

Interferon beta (IFNB) was one of the first approved immunomodulatory treatment for RRMS [23]. Different therapeutic regimens are used, with a subcutaneous administration ranging from three times weekly to twice monthly [23]. For RA, an open phase I study revealed that treatment with IFNB was generally well tolerated, and that a trend toward clinical improvement was observed [24]. In contrast, a phase II study failed to demonstrate the effectiveness of IFNB treatment as no significant differences were found compared with placebo in radiological scores, clinical parameters, or microscopic analysis of synovial tissue samples [25].

The currently available data do not support the use of interferons for the treatment of MS and comorbid RA.

#### Methotrexate and azathioprine

Methotrexate and azathioprine are both approved by the EMA and FDA for the treatment of RA [26]. In the early 2000, both drugs were also investigated in RCT for the treatment of MS [27, 28]. While moderate beneficial effects were observed, particularly in individuals with progressive forms of MS, neither methotrexate nor azathioprine received approval from the EMA or FDA for this indication, although some countries use these substances [27, 28]. Nevertheless, in some European countries, including Germany, and in low-resource settings where access to approved DMT is limited, azathioprine has been used or proposed as an alternative to approved on-label therapies [28]. However, at present, azathioprine and methotrexate hold no significant role in the treatment of MS in the European Union or the US [27, 28].

#### Secukinumab

For RA secukinumab, a monoclonal antibody directed against IL-17, was investigated in three phase III studies. Here, people with RA that did not sufficiently respond to a previous therapy with TNFα inhibitors, secukinumab was superior to placebo in two of the studies (but numerically less efficacious as abatcept, an active comparator) and not superior to placebo in the second study [29]. In a metaanalysis of these studies, secukinumab was significantly better than placebo to achieve a mild improvement of RA activity (ACR20 response; American College of Rheumatology), but was not better than placebo in achieving strong responses (ACR50 and ACR70) [29]. EMA- and FDA-approvals for this indication are therefore not available. Despite this, based on effective clinical trials, secukinumab is approved by the EMA and FDA for the treatment of plaque psoriasis, psoriatic arthritis, and axial spondyloarthritis [30].

Although a phase II study using secukinumab (10 mg/kg i.v.) in people with RRMS reported a significant reduction in the number of new gadolinium-enhancing T1 lesions, there was only a non-significant trend towards reducing the cumulative number of combined unique active lesions on MRI compared to placebo [31]. In contrast, some reports indicate that people with highly active MS may not have responded adequately to the subcutaneous regimen, requiring the continuation of treatments like fingolimod or dimethyl fumarate, or switching to rituximab [32, 33].

In summary, treatment with secukinumab can be offered to people with RA and comorbid MS as an *off label* therapy, particularly in mildly active forms, as some beneficial effects have been observed. However, it is likely that the drug will not be sufficient for highly active forms of MS and RA.

#### Abatacept

Abatacept, a recombinant fusion protein consisting of human IgG and the extracelluar domain of cytotoxic T lymphocyte–associated protein 4 (CTLA4), is approved by both the EMA and FDA for the treatment of adult RA [34].

Despite being investigated in two phase II trials, abatacept did not demonstrate significant treatment benefits in people with MS [35]. The first trial was stopped prematurely due to improper study population acquisition, while the second phase II study showed no efficacy in reducing the number of new gadolinium-enhancing MRI lesions or in clinical measures of disease activity [35].

In summary, abatacept cannot be recommended for the treatment of comorbid MS, as evidence supporting its efficacy in managing MS is lacking. Further studies are necessary to explore its potential in this context.

#### Bruton´s tyrosine kinase inhibitors

Different BTK inhibitors such as ibrutinib and acalabrutinib are approved for the treatment of certain B-cell malignancies (e.g., chronic lymphocytic leukemia) [36]. So far, no BTK inhibitors have been approved by the EMA or FDA for autoimmune diseases such as MS or RA. However, BTK inhibitors are being intensively studied in clinical trials for these indications, since BTK appear in B lymphocytes, myeloid cells, and platelets and play a crucial role in signal transduction via B cell and Fc receptors [37]. Currently, five oral BTK inhibitors are investigated for people with MS: evobrutinib, tolebrutinib, fenebrutinib, remibrutinib and orelabrutinib. For evobrutinib, the phase III trials in people with RMS yielded negative results in reducing relapse rates, demonstrating non-superiority compared to teriflunomide, along with the failure to meet secondary endpoints and concerns regarding liver-related safety issues [38]. Similarly, tolebrutinib did not demonstrate a reduction in relapse rates compared to teriflunomide in people with RMS [39]. However, positive treatment effects have been reported with a reduction in progression, especially in people with progressive forms of MS [40]. For the other currently investigated BTK inhibitors, study reports are awaited.

For the treatment of RA, various reversible and irreversible BTK inhibitors have been studied [31]. Although phase I trials have shown good tolerability of different BTK inhibitors (evobrutinib, spebrutinib, tirabrutinib and fenebrutinib) in people with RA, phase II trials evaluating evobrutinib, spebrutinib, fenebrutinib and acalabrutinib revealed that treatment efficacy was not as strong as expected on several outcome parameters [41, 42]. In addition, a phase II trial was prematurely stopped after a minimal probability of achieving a clinical response was identified [43].

In summary, the currently available data so far do not support the use of BTK inhibitors for the treatment of MS and comorbid RA. However, ongoing studies might provide additional information, possibly offering a therapeutic option for people with progressive forms of MS.

### Treatment options with lack of evidence in MS or RA

#### Sphingosine-1-phosphate (S1P) receptor modulators

S1P receptor modulators exert their immunomodulatory impact by sequestering lymphocytes within secondary lymphoid organs, like lymph nodes, through the degradation of S1P receptors [44–46]. This action leads to a reduction in the number of circulating lymphocytes [44–46]. This mechanism has demonstrated efficacy in the treatment of people with MS by restricting the entry of lymphocytes into the central nervous system (CNS), prompting approvals for fingolimod and ozanimod for RRMS, siponimod for active secondary progressive MS (SPMS), and ponesimod for RMS [44–46]. The prescribed daily maintenance dosages are 0.5 mg for fingolimod, 0.92 mg for ozanimod, 1–2 mg (depending on CYP genotype) for siponimod, and 20 mg for ponesimod [44–46].

To date, clinical trials investigating the use of S1P receptor modulators for RA have not been conducted. Consequently, no recommendation can be made regarding the use of S1P receptor modulators in patients with MS and comorbid RA.

#### Glatiramer acetate

Glatiramer acetate is EMA- and FDA-approved for RRMS and clinically isolated syndrome and is subcutaneously applied daily or three times per week depending on the dosage applied 20 mg, 40 mg) [47, 48]. Our investigation revealed no clinical trials of glatiramer acetate in the treatment of RA. Due to its mechanism of action in MS, it is likely that both outcomes, lack of efficacy or potential negative effects, are possible when using glatiramer acetate, thus the treatment of people with MS and comorbid RA can not be recommended.

#### Janus kinase (JAK) inhibitors

JAK inhibitors are kinases, which primarily act through the JAK/STAT pathway to inhibit cytokine signaling [49]. Tofacitinib, baricitinib, filgotinib and upadacitinib are approved by the EMA and FDA for the treatment of RA [50]. To date, no studies have investigated the use of JAK inhibitors for the treatment of MS in humans. However, in animal models of MS, beneficial treatment results of JAK inhibitors were reported.

In summary, JAK inhibitors are an approved and well-established treatment option for RA. However, clinical trials are necessary to evaluate their potential use in the treatment of MS.

#### Chimeric antigen receptor (CAR) T-cell therapy

CAR T cells are genetically engineered immune cells designed to target and eliminate cells presenting specific surface receptor, such as CD19. This form of immunotherapy involves modifying a patient’s T cells to express a synthetic receptor (CAR) that specifically recognizes target antigens, followed by their reinfusion into the patient. To date, only a few case reports have investigated the treatment with CAR T cells targeting CD19 in people with MS or RA [51, 52]. These reports predominantly describe beneficial treatment effects [51, 52]. However, further studies will be needed to define a patient collective profiting from deep B cell depletion by using CAR T cells.

#### Tocilizumab/sarilumab

Tocilizumab and sarilumab, monoclonal antibodies directed against the IL-6 receptor, have been approved by the EMA and FDA for the treatment of RA [53, 54].

However, no clinical trials have investigated anti-IL-6 receptor antibodies in people with MS. The available case reports on tocilizumab use in MS show conflicting results: two cases involving individuals with fulminant MS and RA suggested beneficial effects, while three cases reported CNS autoimmunity, including the development of new demyelinating lesions and relapse onset associated with its use [55–58]. No data were found on the use of sarilumab in people with MS.

In summary, due to insufficient evidence, treatment with tocilizumab or sarilumab is not recommended for individuals with RA and comorbid MS.

### Treatment options without beneficial effects and reported adverse events in MS or RA

#### Dimethylfumarate (DMF), diroximelfumarate (DRF)

Dimethyl fumarate (DMF) is approved for the treatment of RMS with a maintenance dose of 480 mg/day, while diroximel fumarate (DRF) is approved for RMS with a maintenance dose of 190 mg/day [59, 60]. These medications are believed to exert their effects by activating cytoprotective genetic signaling pathways, leading to immunomodulation [61]. However, due to insignificant changes in endpoint measurements observed in a phase II study for RA, further trials and regulatory approval for this indication were not pursued [62, 63]. Additionally, clinical trials for DRF in RA have not been conducted. Severe adverse reactions to DMF are rare but may include arthritis and polyarthralgia [64].

The currently available data do not support the use of DMF or DRF for the treatment of MS with comorbid RA.

#### Alemtuzumab

Alemtuzumab is a humanised monoclonal anti-CD52-antibody, which leads to a profound and long-lasting lymphopenia due to depletion of T and B cells [65, 66]. Based on effective clinical trials, it is approved as a treatment option for highly active MS [65].

Although alemtuzumab was investigated in two clinical trials for RA, one uncontrolled trial with 40 patients and the other a monocentric trial with 41 patients, approval for this indication was not pursued due to the lack of a striking therapeutic response and the occurrence of fatal opportunistic infections [66, 67].

In summary, alemtuzumab should be used strictly within its approved indications and is recommended only for patients with MS. Its use in individuals with RA is not supported, and current drug information explicitly prohibits its administration to people with comorbid autoimmune diseases.

#### Natalizumab

Natalizumab, which antagonizes alpha-4 (α4) integrin, suppresses lymphocytic invasion in both the CNS and the bowel, leading to its approval as an effective treatment for RRMS by the EMA and FDA, and for Crohn´s disease by the FDA [68]. However, it has not been approved for RA as clinical trials in this area have not been conducted. In addition, a case report described development of RA soon after initiation of natalizumab treatment for MS, and control of symptoms following natalizumab discontinuation and addition of methotrexate [69].

Since no clinical trials are available and adverse events were reported, natalizumab to treat people with MS and comorbid RA cannot be advised.

### Treatment options that should be avoided

#### Tumor necrosis factor alpha (TNFα) inhibitors

TNFα inhibitors and soluble TNFα receptor IgG fusion proteins are EMA- and FDA-approved for the treatment of RA [70]. These therapies are also approved for the treatment of psoriatic arthritis and ankylosing spondylitis. Furthermore, TNFα inhibitors are approved for use in inflammatory bowel diseases, including Crohn’s disease and ulcerative colitis [71].

Based on pathophysiological considerations, infliximab was tested in a phase I study involving two people with MS, which reported the onset of new MRI lesions and pleocytosis in the CSF after each infusion [72]. Despite these findings, a phase II trial investigated lenercept (a TNFα receptor IgG fusion protein) in 168 people with MS [73]. However, the study was prematurely terminated due to a dose-dependent increase in relapse frequency and the accumulation of neurological deficits [73].

A recent meta-analysis reported a similar risk for the occurrence of central nervous system inflammatory disease in different types of underlying autoimmune diseases during TNF-α inhibitor therapy (monoclonal antibodies vs. TNF-α receptor IgG fusion proteins) [7].

In summary, treatment with TNFα inhibitors is contraindicated in people with RA and comorbid MS.

## Discussion

Currently, more than 15 effective therapies for people with MS are available allowing for a more individualized treatment considering specific conditions such as highly or moderately active MS, pregnancy or autoimmune comorbidities and emerging new therapies will provide additional treatment options [6]. Therefore, an overview of the therapeutic effects of various approved and currently investigated MS-DMT in RA and vice versa was provided.

In people with MS and comorbid RA, teriflunomide and its precursor leflunomide, as well as anti-CD20 therapies, are either approved by the EMA and FDA for their respective indications or have demonstrated beneficial treatment effects in phase III studies, making them the treatments of choice [8–19]. Among these options, anti-CD20 therapies should be prioritized for individuals with highly active MS, while teriflunomide is more suitable for those with moderately active form of the disease [8–19]. Although data on RA is limited, oral cladribine provide an additional therapeutic option for people with comorbid MS, owing to their broad mechanism of action, which targets both B and T lymphocytes [20–22]. Especially if anti-CD20 therapies need to be switched, for instance, due to disease activity or hypogammaglobulinemia, cladribine could be considered as a treatment option for people with MS and comorbid RA [20–22, 74]. However, among the many DMT investigated for MS and RA, several compounds failed to demonstrate sufficient treatment efficacy, and thus no phase III studies were conducted. Consequently, abatacept (no effects on MS), IFNB (no effects on RA), azathioprine (insufficient effects on MS), and BTK inhibitors (no effects on RA) are unlikely to provide a meaningful treatment response in people with MS and comorbid RA [23–28, 34–43, 75]. However, BTK inhibitors might play a crucial role in the future for targeting progression in people with MS, encouraging further trials of these compounds in people with RA [40]. Additionally, while currently supported only by one phase II study for MS and different phase III studies for RA, secukinumab could be a therapeutic option for moderately active forms of MS and RA [30–36]. However, secukinumab might not be an adequate treatment for highly active MS and RA forms [29, 31–33].

In contrast, despite their reported efficacy in MS, no clinical trials have been conducted to evaluate S1P receptor modulators or glatiramer acetate efficacy in RA [44–48]. In a similar way, while effective and approved for RA, JAK inhibitors were not evaluated in the context of comorbid MS and RA [48, 49]. CD19 targeting CAR T cell treatment constitutes a novel and innovative treatment method for autoimmune disorders, such as MS and RA [51, 52]. However, due to the few available reports, more studies will be needed to assess to benefit in these patient collectives.

Lastly, there are DMT that should be avoided or are even contraindicated in people with MS and comorbid RA. For example, DMF and alemtuzumab were investigated in phase II trials in people with RA but did not show significant beneficial treatment effects [62–64, 66, 67]. However, severe adverse events of fatal opportunistic infections as well as onset or exacerbation of RA were reported and the label for alemtuzumab was revised to contraindicate its use in individuals with comorbid autoimmune diseases. For tocilizumab (approved for RA) and natalizumab (approved for MS), no clinical trials have been conducted for the other condition, yet adverse events have been reported. In contrast, a phase II trial of lenercept in people with MS demonstrated disease activity clinically and on MRI, leading to its early termination [73]. Therefore, TNFα inhibitors are contraindicated in people with MS.

However, the data presented in this review article is not free of limitations. Interpreting this data the overall low number of cases and the lack of prospective studies for individual substances has to be considered.

## Conclusion

Of the many approved DMT for MS and RA, teriflunomide/leflunomide and anti-CD20 therapies represent the most suitable treatment options for people with moderately or highly active MS, respectively, and comorbid RA. While oral cladribine may also be considered as a treatment option for this population, TNFα inhibitors are contraindicated.

## Data Availability

The datasets used and/or analysed during the current study are available from the corresponding author on reasonable request.
